# Vestibular Perception following Acute Unilateral Vestibular Lesions

**DOI:** 10.1371/journal.pone.0061862

**Published:** 2013-05-09

**Authors:** Sian Cousins, Diego Kaski, Nicholas Cutfield, Barry Seemungal, John F. Golding, Michael Gresty, Stefan Glasauer, Adolfo M. Bronstein

**Affiliations:** 1 Neuro-otology Unit, Division of Brain Sciences, Imperial College London, Charing Cross Hospital, London, United Kingdom; 2 Neurology, Dunedin Hospital, University of Otago, Dunedin, New Zealand; 3 Department of Psychology, University of Westminster, London, United Kingdom; 4 Sensorimotor Research and German Vertigo Center, Ludwig-Maximilian University, Munich, Germany; Emory University, United States of America

## Abstract

Little is known about the vestibulo-perceptual (VP) system, particularly after a unilateral vestibular lesion. We investigated vestibulo-ocular (VO) and VP function in 25 patients with vestibular neuritis (VN) acutely (2 days after onset) and after compensation (recovery phase, 10 weeks). Since the effect of VN on reflex and perceptual function may differ at threshold and supra-threshold acceleration levels, we used two stimulus intensities, acceleration steps of 0.5°/s^2^ and velocity steps of 90°/s (acceleration 180°/s^2^). We hypothesised that the vestibular lesion or the compensatory processes could dissociate VO and VP function, particularly if the acute vertiginous sensation interferes with the perceptual tasks. Both in acute and recovery phases, VO and VP thresholds increased, particularly during ipsilesional rotations. In signal detection theory this indicates that signals from the healthy and affected side are still fused, but result in asymmetric thresholds due to a lesion-induced bias. The normal pattern whereby VP thresholds are higher than VO thresholds was preserved, indicating that any ‘perceptual noise’ added by the vertigo does not disrupt the cognitive decision-making processes inherent to the perceptual task. Overall, the parallel findings in VO and VP thresholds imply little or no additional cortical processing and suggest that vestibular thresholds essentially reflect the sensitivity of the fused peripheral receptors. In contrast, a significant VO-VP dissociation for supra-threshold stimuli was found. Acutely, time constants and duration of the VO and VP responses were reduced – asymmetrically for VO, as expected, but surprisingly symmetrical for perception. At recovery, VP responses normalised but VO responses remained shortened and asymmetric. Thus, unlike threshold data, supra-threshold responses show considerable VO-VP dissociation indicative of additional, higher-order processing of vestibular signals. We provide evidence of perceptual processes (ultimately cortical) participating in vestibular compensation, suppressing asymmetry acutely in unilateral vestibular lesions.

## Introduction

The vestibulo-ocular reflex (VOR) has been extensively investigated in health and disease [1]. Comparatively little is known about the functional properties of the vestibulo-perceptual (VP) system, particularly following an acute peripheral unilateral vestibular lesion. Although their precise distribution remains obscure [2], cortical vestibular networks presumably mediate perception of whole-body motion. As with all psychophysical systems, decisions (“in which direction am I moving?”) are based on the detection of signals (stimuli), such as semicircular canal afferent information, against a background of activity, or *noise*. Vestibular afferents transmit signals to the brainstem for VOR control and further to the cortex for conscious perception [3] but one question is, to what extent is this signal modified at the cortical level in disease? As with other psychophysical systems, the VP system has a defined threshold at which a signal becomes recognised relative to the noise level [4], [5]. For the vestibular system, humans possess higher perceptual than VOR thresholds [6]. These higher perceptual thresholds in humans probably reflect the cortical processing of vestibular signals during decision making in direction recognition tasks [7].

Significant cortical changes are observed in patients following acute vestibular lesions [8], [9]. Conventional vestibular testing, however, focuses on reflex responses and therefore provides little insight into the functioning of the substantial vestibulo-cortical projection – responsible for sensations of rotation [10] and hence vertigo [11]. Given that dizziness and vertigo are *percepts*, the dearth of papers on this topic is surprising. Thus, we investigated whether a peripheral vestibular lesion alters low-level (VO) and high level (VP) vestibular processing similarly or whether the presence of the strong rotational vertigo experienced after vestibular lesions specifically disrupts vestibulo-perceptual (presumably cortical) processing. The presence of such a vigorous symptom could impair threshold function (e.g. recognising the direction of whole-body motion) or interfere with the velocity storage system at a perceptual level [10], [12], [13]. Thus, we hypothesised that the acute vestibular lesion could disrupt the harmonious relationship present between VO and VP function and dissociate the responses of these two systems. In addition, examining patients with unilateral lesions allows us to pose questions on low-level vestibular function such as, is a single labyrinth capable of detecting motion at normal threshold levels in both directions?

To explore these effects, we simultaneously investigated VO and VP function in 25 patients with vestibular neuritis (VN) in the acute phase and after compensation. Given that the dynamic properties of the vestibular system change at threshold and supra-threshold acceleration levels [14], we examined the function of these two vestibular projections appropriately at two stimulus intensities.

## Materials and Methods

### Subjects and methods

Twenty-five patients (mean age 46 years, sd 15.68, 13 females) were studied in the acute (1–5 days after vertigo onset, median 2 days) and recovery (6–16 weeks, median 10 weeks) phases of VN. Acutely, clinical examination revealed unidirectional horizontal nystagmus with a slight torsional component, a positive horizontal head impulse test, unilateral canal paresis on caloric testing (20%, [15]), unsteadiness and no hearing impairment. There were no symptoms/signs of CNS disorder. Twenty-four patients received antiemetic medication (prochlorperazine/cyclizine) in the acute stage, for nausea and vomiting, but only three patients received medication on the day of testing. No patient received corticosteroids. Acutely, all patients were bedridden due to vertigo symptoms, improving to normal or near normal levels of activity at the recovery stage. At each stage patients underwent vestibular caloric testing and clinical assessment in addition to threshold and supra-threshold psychophysical angular velocity vestibular tasks. Thirty normal subjects were recruited as controls (mean age 42 years, sd 18.1, 15 females).

#### Ethics Statement

Individual informed written consent was obtained from all subjects and the study was approved by Charing Cross Hospital Research Ethics Committee.

### Psychophysical angular velocity tasks

**Figure 1:**
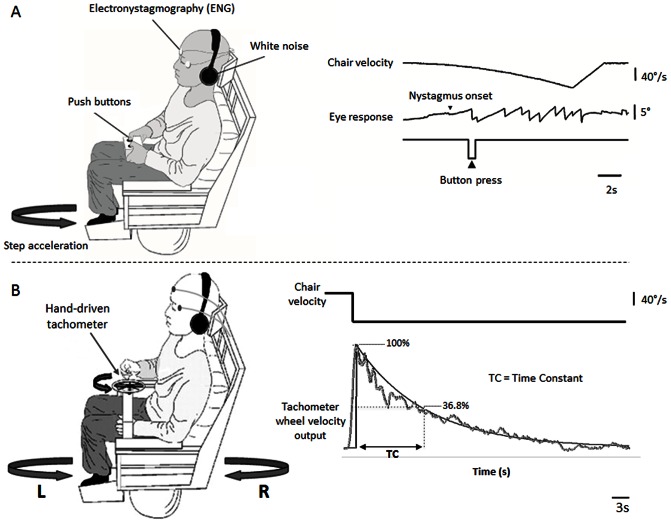
Schematic diagram showing a subject sitting on the rotating chair for simultaneous psychophysical and eye movement (ENG) assessment in the dark. A: Threshold vestibular task. The subject carries a hand-held device with two buttons (left and right) whilst exposed to step acceleration rotations with an initial acceleration of 0.5°/s^2^, increasing by 0.5°/s^2^ every 3 s. The subject presses the appropriate button to indicate perceived direction (leftward vs. rightward) as soon as they were sure they were moving in a particular direction. Vestibulo-perceptual (VP) thresholds were measured by the time taken from chair acceleration onset to button press (button press) and converted to °/s when appropriate. The vestibulo-ocular (VO) threshold was measured as the point at which the slow-phase eye velocity curve left the baseline and did not return (nystagmus onset). B: Supra-threshold vestibular task. Subjects in the motorised rotating chair were exposed to a velocity step of 90°/s for 60 s, either leftwards or rightwards. They were instructed to turn the wheel at maximal speed on starting/stopping rotation (the point of maximal subjective and ocular angular velocity) and to slow the tachometer speed in proportion to their own perceived slowing of rotational velocity [10]. A representative raw trace and fitted exponential curve from the tachometer wheel in a normal subject is shown on the right. This allows for accurate measurement of the time constant (TC) of decay of the vestibular perceptual response. Vestibulo-ocular responses (not shown) were obtained using electronystagmography (ENG = EOG), and follow a similar exponential decay to the perceptual responses.

Figure 1 shows the experimental set-up for both threshold and supra-threshold angular velocity tests. Rotations were performed using a vibration-free motorised rotating chair (Contraves, USA), with sound masking to eliminate non-vestibular cues. Adjustable chin and head rests minimised head movements. Both tests were carried out in total darkness - subjects were surrounded by a 360° black out curtain in a windowless custom built vestibular darkroom. Vestibulo-ocular (VO) responses were recorded with bi-temporal electro-oculography (EOG) from adhesive electrodes on the outer canthi of the eyes.

#### Threshold vestibular task (Figure 1A)

This test simultaneously measures vestibulo-perceptual (VP) and vestibulo-ocular (VO) thresholds for detection of angular motion. Subjects were seated in the rotating chair with a hand-held device with two buttons. The test comprised 3 rightward and 3 leftward rotations, with an initial acceleration of 0.5°/s^2^, increasing by 0.5°/s^2^ every 3 s. The instruction was to press the appropriate button to indicate perceived direction (leftward vs. rightward) as soon as they were sure they were moving in a particular direction. The incremental acceleration continued until the subject indicated their perceived direction of rotation or, if there was no response, up to a maximum velocity of 82.5°/s at 30 s. Vestibulo-perceptual thresholds were measured by the time taken from chair acceleration onset to button press. The vestibulo-ocular threshold was measured as the point at which the slow-phase eye velocity curve left the baseline and did not return (details in [16]). Any resting spontaneous nystagmus is included in baseline responses and thus thresholds are taken as the point at which the spontaneous nystagmus is modified by the rotational stimulus. If the subject failed to perceive a rotation or if there was no change in slow phase eye velocity, then a threshold of maximum trial duration was taken (30 s). If a subject's thresholds were bilaterally higher than could be measured within the limits of the test (i.e. not reached in 30 s at a velocity of 82.5°/s) they were excluded from asymmetry analysis. If a subject reported perceived rotation in the wrong direction (e.g. pressing the right button during a leftward rotation), the incorrect button push was noted and the rotation repeated. If the subject continued to press the incorrect button on the repeat rotation the trial was excluded from analysis. Both VO and VP thresholds were measured for each trial and a median taken for rotations towards the healthy side (contralesional) and the affected side (ipsilesional). The mean and standard error of these median values are presented in results. The median was used as a representative value because there were only three rotations per side. However, calculation of a mean value per subject and direction showed an almost identical value (see Results, under Threshold vestibular function).

#### Supra-threshold vestibular task (Figure 1B)

Vestibulo-ocular and vestibulo-perceptual responses were measured following eight +/−90°/s velocity steps (starting-stopping Barany rotational test), lasting 60 s with acceleration/deceleration phases of 1 s.

Perceptual responses were measured by subjects turning a tachometer wheel after the accelerations/decelerations to give an analogue indication of their perceived rotational velocity. Subjects were instructed to turn the wheel at maximal speed on starting/stopping rotation (the point of maximal subjective and ocular angular velocity) and to slow the tachometer speed in proportion to their own perceived slowing of rotational velocity [10]. The tachometer output follows an approximately exponential decay allowing accurate measurement of the time constant of decay of the vestibular perceptual response (normal subjects mean R^2^ = 0.95). An additional measurement, independent of the decay function, is the duration of the tachometer-wheel trace. Time constant and duration measurements of amplitude-normalised VOR responses were taken for comparison. An exponential curve was fitted to the decay portion of both perceptual and vestibulo-ocular velocity outputs (peak response to return to baseline) and the dominant time constant was derived from a maximised R^2^ goodness of fit value. Duration was calculated as time taken from initial onset of response to cessation of wheel turning (no sensation) and a return to the eye velocity baseline values. As with the threshold analysis, spontaneous nystagmus slow phase velocity was included in the baseline and thus *de facto* excluded from measurements. The procedure feels natural and intuitive to the subjects and it has been validated in normal and patient studies [10], [12], [13], [17]–[19].

### Caloric and rotational responses

Bithermal (30 and 44°C) caloric stimulation was carried out and degree of canal paresis [CP; Jongkees formula−right warm slow phase velocity (SPV)+right cold SPV)−(left warm SPV+right cold SPV)/(right warm+right cold+left warm+left cold SPV)*100] and average caloric function (average peak SPV across all caloric irrigations) measured. During the 90°/s velocity steps, VOR gain (peak slow-phase eye velocity/peak chair velocity) was also measured.

### Statistical analysis

Patient responses were compared to control subjects using one-way ANOVAs for threshold and supra-threshold tasks. Normal control responses to right and left rotations were compared to patients' contralesional and ipsilesional responses, respectively.

Repeated measures 2×2 ANOVA's were carried out for threshold and supra-threshold tests with factors, Response type (vestibulo-ocular vs. vestibulo-perception), Rotation direction (contralesional vs. ipsilesional) and between subjects factor, Subject group (patients vs. normals). In five patients both contralesional and ipsilesional thresholds (n = 3 VO, n = 2 VP) were bilaterally higher than could be measured within the limits of the vestibular threshold test (i.e. above 82.5°/s) and so were excluded from this 2×2 ANOVA asymmetry analysis. Threshold data is presented in both the raw recorded units (seconds) and velocity (degrees per second).

Supra-threshold duration measurements correlate well with time constant (r = 0.8, P<0.001) measurements, and so were used as the primary measurement in correlational analysis comparing VO and VP responses across threshold and supra-threshold tasks. In order to investigate any association between VO/VP results from the psychophysical tasks and conventional vestibular function measures, we correlated VO/VP thresholds and supra-threshold results with VOR gain, degree of spontaneous nystagmus and caloric canal paresis. Patients were also split into two groups on the basis of the severity of the canal paresis (range 20–100%, allowing for the CP>20% inclusion criteria). Thus, two groups of VN patients were produced and compared to normal subjects - those in the top third percentile (severe CP>73%, n = 13) and those in the two lower percentiles (moderate CP = 20–72%, n = 12). Results are reported as significant at the p<0.05 level with Bonferroni correction used for multiple correlations. Outliers were defined as any data point more than 1.5 times the interquartile range below quartile 1 or above quartile 3 and were identified automatically by SPSS (Version 18).

### Sensor fusion and signal detection model

**Figure 2:**
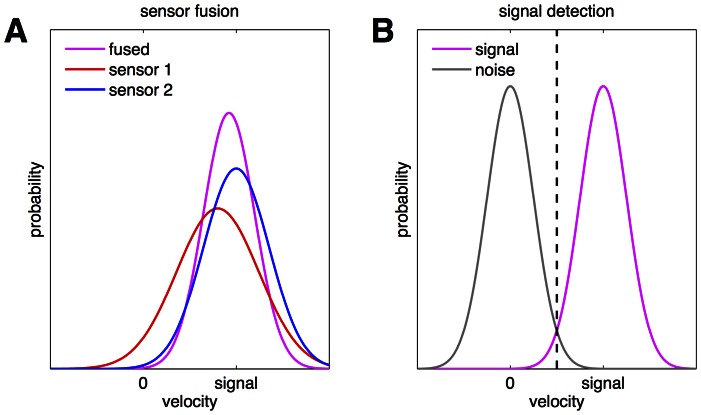
Schematic illustration of the sensor fusion and signal detection model. A: Sensor fusion as maximum-likelihood estimation: the two likelihood functions (red and blue distributions) for two sensors with slightly different sensor variability and gain are shown. The blue distribution is correctly centred at the signal, while the red, more variable distribution is centred closer to zero. The pink curve shows the combined distribution, which has a lower variability. B: Signal detection: the combined signal distribution (pink) and the noise distribution (centred at zero) are assumed to have equal variance. The threshold signal is reached, if the overlap between both distributions becomes small enough. The respective decision criterion is marked by the dashed line.

As mentioned in Introduction, threshold measurements determine at which level a signal can be differentiated from background noise. This corresponds to a standard signal detection task, which can be quantitatively modelled by signal detection theory [4], [5], [20]. According to this theory, the threshold is reached when the distance between signal distribution (centred at the signal) and noise distribution (centred at zero) exceeds a certain distance (Figure 2B), which depends on the signal variability. For the present case, we assumed the standard model of Gaussian distributions with equal standard deviation for signal and noise distributions [20]. We further assume that the decision criterion (the probability for correct response) remains constant, i.e. that for a given detection task, the required separation of the signal from the background noise is of the same size for acute and recovery stages. The model was applied to VO and VP thresholds.

To model the interaction between both labyrinths, we assume that signals from the left and right labyrinth are fused to yield a central estimate of head rotation. A probabilistic estimation taking into account the noise of the fused signals leads to the maximum-likelihood strategy (Figure 2A), which, for Gaussian noise, results in weighted averaging (eg., [21]). Accordingly, if the signal on each side is normally distributed with variance σ^2^, the fused signal will become more accurate with smaller variance σ^2^/2.

If one side is partially lesioned, part of the neural input is missing, which leads to a decrease in baseline firing rate *b* and in transduction gain (see Appendix S1). That is, if the healthy side normally responds to a velocity stimulus *s* with firing rate *f*(*s*) = *b*+*s* (we assume a unity gain for simplicity) then after the partial lesion it will respond with a lower firing rate given by *f_l_*(*s*) = (1−*c*)·(*b*+*s*), with *c* being the amount of the lesion (c = 1 indicates a complete lesion so that the firing rate drops to zero; *c* = 0 is the healthy case). However, the lesion has also another effect: due to the lower number of afferent fibres, the variability of the central response increases. For an acute lesion, we assume that the central fusion mechanism does not yet compensate for the loss in baseline firing (causing vertigo and spontaneous nystagmus), the drop in gain (leading to decreased sensitivity) and the increased variability. In other words, the central fusion mechanism is falsely treating the decrease in firing as stimulus. Therefore, we assume that in the acute case the equal weighting of the ipsi- and contralateral afferent information is retained. Only after recovery, which leads to (central) recalibration of baseline firing and gain [22], the increased variability of the lesioned side is taken into account by the central fusion, which then leads to reduced weighting of the ipsilesional side due to the its increased variability.

However, since patients are never measured immediately after the lesion, one can assume that the recovery process has already started and that, for example, the baseline firing may be partly restored while the gain is still low. From these assumptions, we can calculate the fused central response for the acute case (see Appendix S1). After recovery, we assume that the partially lesioned side is recalibrated and gain and baseline are restored. Thus, it responds like the healthy side, but its variability remains increased, because the missing nerve fibres are not restored. We can thus determine the threshold values for the normal case, the acute lesion and recovery (see Appendix S1).

Our combined model for the healthy case and the recovery phase has only three unknown variables: the decision factor determining the required separation of signal and noise (which is different for perceptual and VO thresholds), the signal variability, and lesion magnitude. Acutely, an additional unknown variable comes into play: the asymmetry in baseline firing. Note that measured spontaneous nystagmus slow phase velocity is not equal to this asymmetry, because the constant afferent bias is amplified by velocity storage. Note also that the present model does not necessarily hold for larger stimuli that may drive one side into saturation. However, since the model is meant to describe the central fusion and decision mechanism for threshold detection, i.e. close to zero stimulation, one can neglect the small effects of non-linearity in the neural activation function of peripheral afferent fibres [23].

Irrespective of the exact values of the free parameters, the most important predictions from the model are (Eqn. 5, Appendix S1): 1) *raised asymmetric thresholds* in the acute stage and 2) *symmetric but elevated thresholds* after recovery. Further predictions for the relation between lesion magnitude and thresholds can be derived from the considerations above (see Results).

Since the model operates on firing rates proportional to angular velocity but experimental threshold values were given in seconds, all simulated responses were converted from deg/s to seconds using the experimental protocol. Numerical simulations were performed using Matlab (Mathworks, Natick, US).

## Results

### Clinical vestibular testing

Acutely, patients had an average canal paresis of 62.53% which improved at recovery stage to 40.4%. In agreement, average caloric responses increased from 16.34°/s acutely to 23.44°/s at recovery stage. A clinically positive head impulse tests was present in only 9 patients at recovery (present initially in all patients). Acutely, VOR gain contralesionally was 0.51, compared to 0.31 ipsilesionally. At recovery VOR gain was similar for rotations towards the affected and the healthy side (contralesional/ipsilesional VOR gain = 0.41). Spontaneous nystagmus in the dark also reduced from an average of 10.16°/s acutely, to 2.38°/s at recovery.

### Psychophysical results

A brief summary will first be presented followed by detailed analysis of all findings.

Acutely, VO and VP thresholds show similar patterns of results and are asymmetrically raised, with ipsilesional thresholds higher than those contralesionally. At recovery, there is a reduction in asymmetry of threshold responses, however in patients with persisting canal paresis both contralesional and ipsilesional thresholds remain raised. The results are well captured by the predictions of the sensor fusion and signal detection model.

Vestibular supra-threshold results (duration/time constants), however, show dissociation between VO and VP responses acutely. Whereas VO time constants are asymmetrically reduced, perceptual responses are suppressed symmetrically.

### Threshold vestibular function

#### Acute stage

Patients detected the direction of motion appropriately. In the acute stage only, two patients pressed the incorrect button on one trial but on repetition pressed the correct button corresponding to the rotation direction. A third patient consistently pressed an incorrect button and these trials were excluded (see Methods). No patient pressed the button in a random fashion.

**Figure 3:**
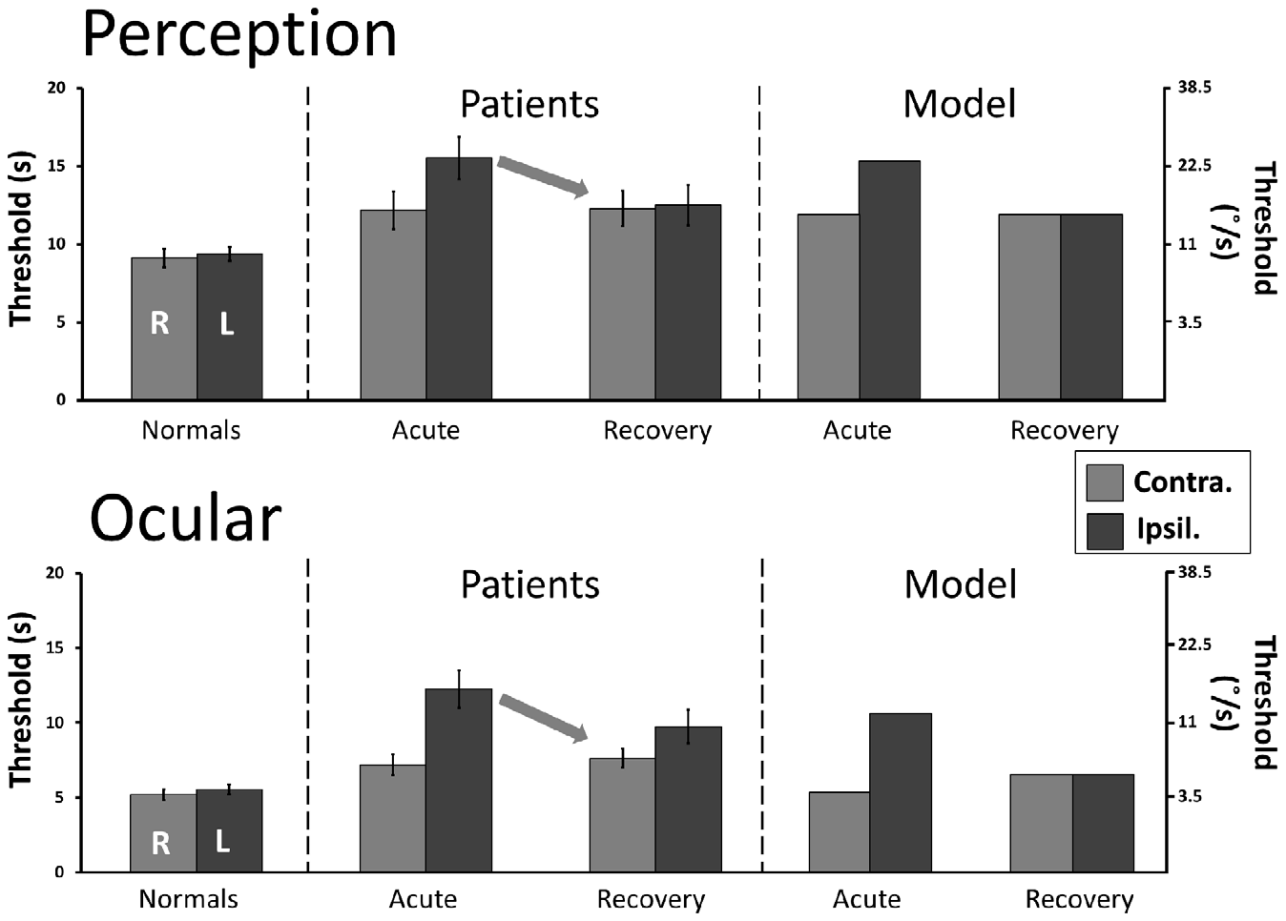
VO and VP thresholds for patients and simulated model data. Mean perceptual (top) and vestibulo-ocular (bottom) thresholds for patient (±SE) and simulated model data, and normal subjects. Thresholds are symmetrical in healthy subjects and lower for VOR than for perception. After acute unilateral vestibular neuritis, thresholds become asymmetric and bilaterally increased with higher thresholds on the affected (ipsilesional) side. After recovery, thresholds remain elevated, but become symmetric again. Simulated thresholds derived from the model show good agreement with patient data; both VO and VP thresholds are elevated and asymmetrical acutely, with a reduction in asymmetry at recovery.

Figure 3 summarises vestibular thresholds results showing, acutely, asymmetrically raised thresholds for both vestibulo-ocular and vestibulo-perceptual responses. The figure shows average median values but mean values were almost identical - the largest difference found between these two measures was, average median = 12.26, average mean = 11.79 for ipsilesional VO thresholds. Intra-subject threshold variability was also increased in patients; the normal subjects VP average range was 8.19°/s and in patients 22.2°/s contralesionally and 29.65°/s ipsilesionally. For VO thresholds, normal subjects average range was 3.42°/s and for patients 10.35°/s contralesionally and 18.5°/s ipsilesionally.

Patient thresholds for rotations towards the affected side are significantly raised above normal for both VP [F(1,53) = 27.77, p<0.001] and VO [F(1,50) = 34.89, p<0.001]. During rotation towards the healthy side, variability is high so that the significant increase in patient thresholds observed [VP, F(1,53) = 8.85, p = 0.004; VO, F(1,50) = 8.4, p = 0.006) disappears with SPSS exclusion of three statistical outliers.

Ipsi-contralesional thresholds are significantly asymmetric (F(1,47) = 30.19, p<0.001), similarly so for VO and VP (i.e. no interaction between VO-VP x ipsi-contralesional thresholds).

The severity of the canal paresis (CP) did not influence the results significantly. The significant difference between acute patients and normals was present in those patients with CP>73% and in those with CP between 20–73%.

#### Recovery stage

As shown in Figure 3 (patient data), overall vestibular thresholds decrease towards normal levels at recovery stage, with a reduction in asymmetry. Intra-individual variability in thresholds across the three trials also lessens at the recovery stage and average ranges decrease towards normal levels (VP: contralesional 19.98°/s, ipsilesional 15.4°/s; VO: contralesional 10.46°/s, ipsilesional 13.02°/s).

In contrast to acute threshold results which are similar across patients regardless of CP, at the recovery stage, patient VO and VP thresholds differ in comparison to normal's based on degree of remaining CP. In patients with persisting canal paresis (>73%) both contralesional [VO, F(1,35) = 10.89, p = 0.002; VP, F(1,35) = 17.11, p<0.001) and ipsilesional [VO, F(1,35) = 40.4, p<0.001; VP, F(1,35) = 23.75, p<0.001] thresholds remain abnormally elevated. In patients with CP>73% there was no significant recovery in thresholds from acute to recovery stages. In patients with CP<73%, both VO and VP thresholds normalise bilaterally (excluding automatically identified outliers, VO n = 2 and VP n = 1).

At recovery, there is a clear reduction in asymmetry between contralesional/ipsilesional thresholds for both VP and VO responses (Figure 3). Indeed, with the two outliers removed there is no significant difference between contralesional-ipsilesional threshold values.

As in normal subjects in this and previous studies [6], VP thresholds were significantly higher than VO thresholds both acutely [F(1,47) = 93.47, p<0.001] and at recovery [F(1,52) = 51.45, p<0.001]. Patient VP thresholds were 28.2% higher than VO thresholds acutely, compared to a 27.5% difference between normal VO-VP thresholds. At recovery the difference between patient VO-VP thresholds slightly increased to 35.21%, however within the normal range. Although overall patient group results show similar findings for both VO and VP thresholds, individual intra-patient correlations between VO and VP thresholds were either weakly or not significantly correlated (all r's = 0.29–0.51, p's = 0.01–0.26, median r = 0.32, p = 0.16), in line with similar observations in normal subjects [6].

#### Model simulation

The signal detection model has four unknown variables: the decision factor *λ*, the signal standard deviation σ, the amount of lesion *c*, and the decrease in ipsilesional baseline firing β (see Methods). In the following, we assume that the decision factor is the same in all cases and that the amount of lesion can be approximated by the acute canal paresis (CP) value. The spontaneous nystagmus slow-phase velocity is assumed to be proportional to the loss in ipsilesional baseline firing (see Methods).

Using these assumptions, we can predict threshold values for an acute lesion and the subsequent recovery from the average acute CP value (63%) and the average healthy threshold responses (Equations 5a and 5b, Appendix S1). For the quantitative prediction, the average VP threshold of healthy subjects (9.2 s), the average VO threshold of healthy subjects (5.25 s), and the average CP acutely (c = 0.63) was used. For the residual baseline firing, we used a fraction (1/4) of the average spontaneous nystagmus (10.2°/s) as estimate for β. Note that the only difference between VO and VP simulation is the amount of the healthy thresholds derived from the data.

The predictions are shown in Figure 3 (alongside patient data). While the perceptual threshold predictions match well, the VO threshold predictions are somewhat low, but also show the expected asymmetry and elevated recovery threshold.

From the model equations (see Appendix S1), individual predictions are also possible. From the individual acute ipsi- and contralesional thresholds *s_ti_* and *s_tc_* and CP value *c*, the individual recovery threshold *s_tr_* can be predicted as *s_tr_* = sqrt(1−*c*/2)·(*s_ti_*+*s_tc_*)/2. This prediction shows a good correlation to the actual recovery threshold for both VP (r = 0.56, p = 0.006) and VO (r = 0.54, p = 0.016). Note that the main factor influencing this prediction is the individual acute threshold. Indeed, the correlation between acute and recovery threshold is already significant for perception (p = 0.012) and VO (p = 0.048). Thus, even though the individual CP factor has only a minor scaling influence reaching from 0.71 to 1.0 (*c* = 1, complete lesion) it critically improves the recovery prediction.

### Supra-threshold vestibular function

#### Acute stage

**Figure 4:**
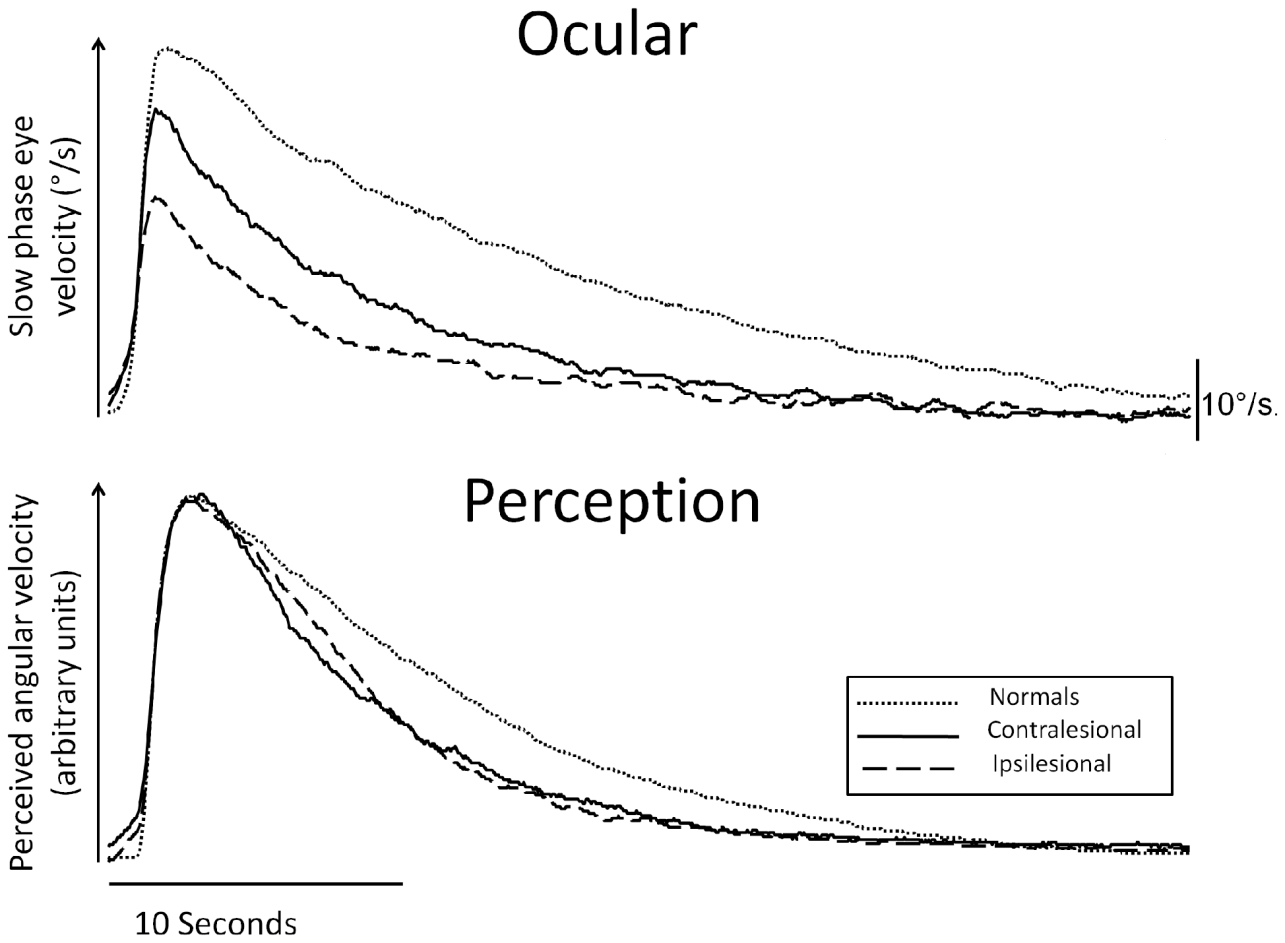
Grand average acute VO and VP responses during supra-threshold task. Grand averages of slow phase eye velocity (vestibulo-ocular) and perceived angular velocity (perception, normalised) in response to 90°/s velocity steps, for normal controls (dotted line) and acute VN patients when accelerating towards the side of the lesion (ipsilesional, dashed line) and towards the healthy side (contralesional, solid line). Note symmetrical and shorter time constants for perceptual data despite grossly asymmetrical ocular responses in acute VN patients.

Responses to 90°/s velocity steps are shown in Figure 4 as grand averages, showing approximately exponentially decaying velocity perception and slow-phase eye velocity. Acute supra-threshold results are significantly reduced for both VO and VP in patients (all ipsilesional and contralesional time constants and durations shortened, p≤0.003). This finding was apparent regardless of degree of canal paresis.

**Figure 5:**
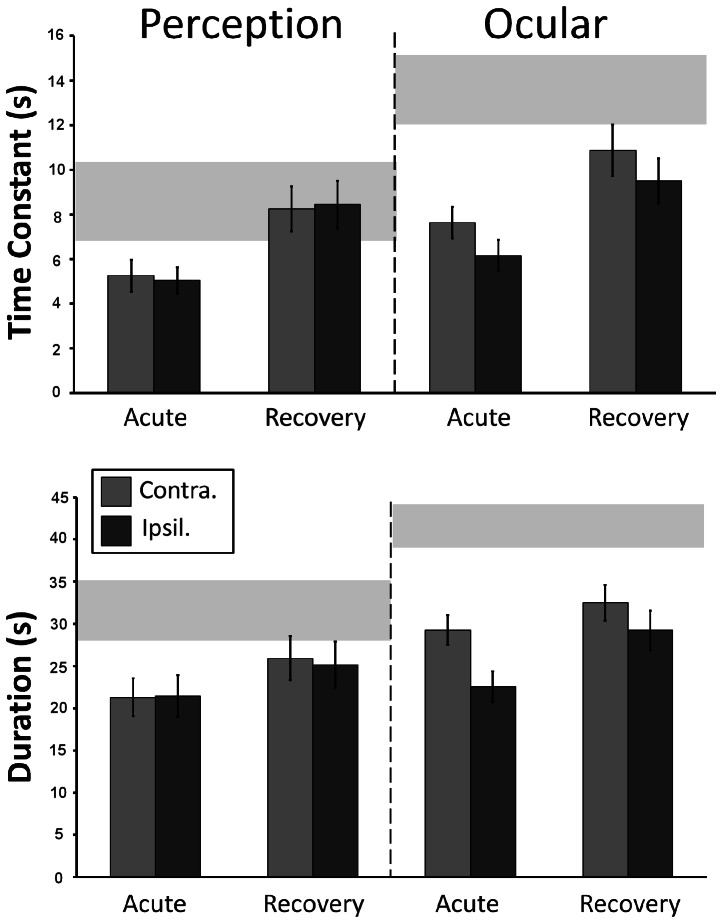
Time constant and duration VO and VP supra-threshold responses at acute and recovery stages. Mean (±SE) supra-threshold duration and time constants for perception (right panel) and vestibulo-ocular (left panel) responses in VN patients, acutely and at recovery. Grey horizontal bars show normative data (95% confidence interval for mean).

Main effects indicate that VO responses are longer than VP [time constant – F(1,51) = 19.05, p<0.001; duration – F(1,51) = 31.2, p<0.001]. Also, a significant asymmetry between contralesional and ipsilesional responses was present [time constant – F(1,51) = 4.85, p = 0.032; duration – F(1,51) = 10.94, p = 0.002]. Of note, however, a significant interaction indicates that the degree of asymmetry differs between VO vs. VP responses of patients and normals [time constant F(1,51) = 6.41, p = 0.015; duration F(1,51) = 9.66, p = 0.003]. Thus, in patients VO responses are reduced and asymmetric but VP responses are further reduced and symmetric (Figure 5, acute).

#### Recovery stage

Figure 5 shows change in supra-threshold responses from acute to recovery stages. At recovery, supra-threshold VP responses have normalised but VO responses remain bilaterally shorter than normal [time constant – contralesional, F(1,52) = 6.45, p<0.014, ipsilesional, F(1,55) = 12.76, p = 0.001; duration – contralesional, F(1,52) = 15.21, p<0.001, ipsilesional, F(1,52) = 23.2, p<0.001], regardless of remaining canal paresis.

Significant contralesional versus ipsilesional [time constants -F(1,52) = 4.36, p = 0.044; duration - F(1,52) = 7.77, p = 0.007] and VO vs. VP [time constants - F(1,52) = 13.51, p = 0.001; duration - F(1,52) = 17.64, p<0.001] main effects remain. Significant interactions persist [time constant F(1,52) = 6.92, p = 0.011; duration F(1,52) = 4.22, p = 0.045], reflecting the continuing symmetrical/asymmetrical nature of VP/VO responses in patients.

### Correlations between VOR gain, caloric and spontaneous nystagmus results, and experimental VO/VP variables

**Figure 6:**
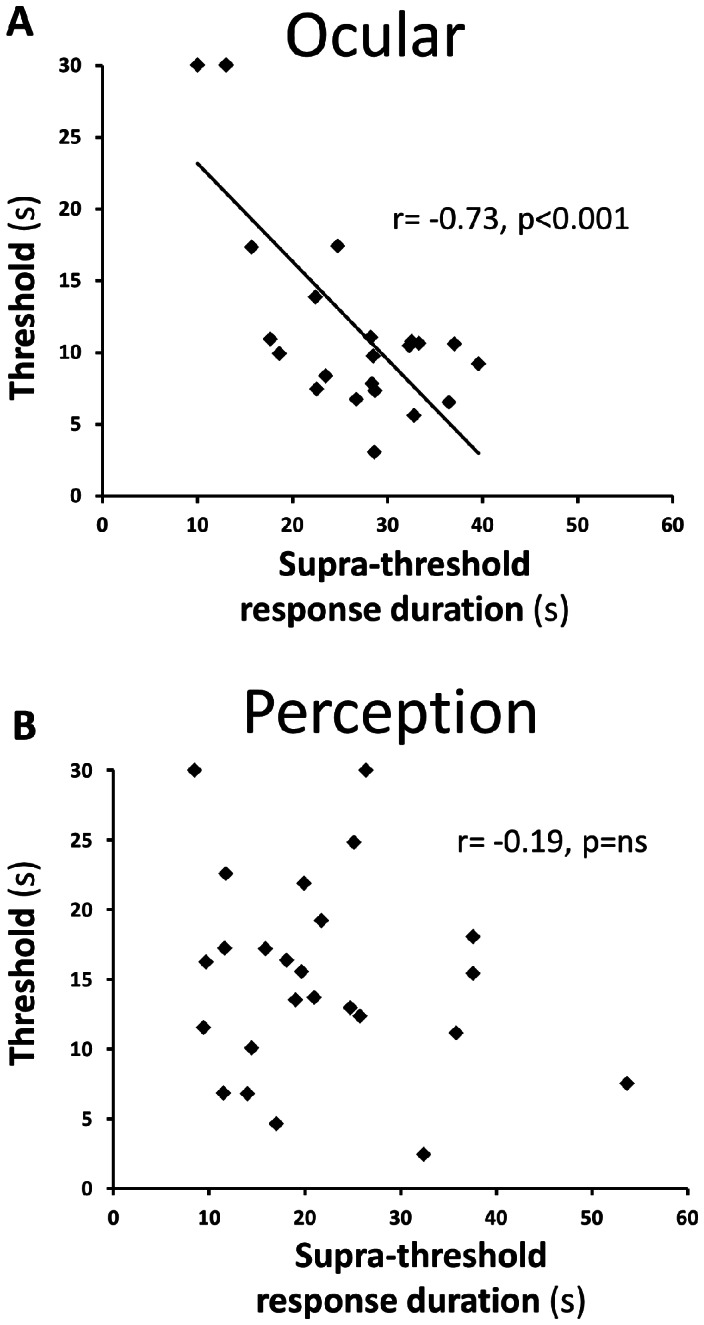
Scatter plots showing correlation between VO/VP threshold and supra-threshold responses. Correlation plots between duration of the response to the supra-threshold stimulus (90°/s velocity step, x axis) and vestibular thresholds (y axis) for the vestibulo-ocular (A) and vestibulo-perceptual systems (B). The plots show good correlation between the two vestibulo-ocular results but absence of correlation between the perceptual results.

Figure 6 shows correlations between threshold and supra-threshold VO and VP responses. Acutely, VO thresholds correlate negatively with VO supra-threshold responses (r = −0.73, p<0.001, Figure 6A), in contrast to VP threshold and supra-threshold results which do not (Figure 6B). There is no association between threshold and supra-threshold VO/VP responses at recovery.

Vestibular threshold function: Caloric findings show some correlation with thresholds at recovery only (VO: ipsilesional r = 0.67, p = 0.001; VP: ipsilesional - r = 0.55, p<0.001, contralesional r = 0.51, p = 0.016). Spontaneous nystagmus does not correlate with thresholds acutely, although there is correlation with contralesional perceptual thresholds at recovery only (r = 0.55, p = 0.01). There is no correlation between thresholds and VOR gain acutely or at recovery

Vestibular supra-threshold function: VO supra-threshold results show no correlation with spontaneous nystagmus acutely or at recovery, although VO supra-threshold responses do correlate with VOR gain and CP (Acute: VOR gain - r = 0.68, p<0.001; Recovery: VOR gain - contralesional, r = 0.71, p<0.001, ipsilesional, r = 0.61, p = 0.002; CP contralesional, r = −0.6, p = 0.002; ipsilesional, r = −0.52, p = 0.01). Of note, however, supra-threshold VP responses do not correlate with VOR gain or CP.

## Discussion

We investigated threshold and supra-threshold vestibulo-ocular (VO) and vestibulo-perceptual (VP) function in 25 patients in the acute and recovery stages of VN.

Firstly, we report that VO and VP thresholds show similar patterns of response in the acute stage after VN. Both VO and VP thresholds are abnormally, asymmetrically raised, with thresholds towards the affected side significantly higher than those towards the healthy side. The balanced discharge rate of vestibular nuclei neurons at rest allows bidirectional modulation of activity in response to movement; this facilitates detection of acceleration in both ‘on’ (excitatory) and ‘off’ (inhibitory) directions [24], [25]. The threshold asymmetry observed in acute VN indicates that the loss of ipsilesional discharge at rest affects threshold detection, a finding replicated by our signal detection model. The resulting unilateral imbalance in resting discharge creates an offset in the central fusion of vestibular signals from both sides leading to asymmetric thresholds. As expected, previous studies have found an overall increase in thresholds in bilateral vestibular failure [16], [26], [27]. The raised thresholds observed in the acute and chronic (in those patients with persisting severe canal paresis) stages reflects the loss of one labyrinthine input, which increases the uncertainty of the rotational velocity input signal (shown also by the increase in individual subjects' VO and VP threshold variability acutely): i.e. with only one functioning labyrinth the signal-to-noise ratio of the velocity signal drops and therefore low velocity rotations are more difficult to detect against background neural noise.

Increase of ipsilesional thresholds in acute VN thus reflects, just as the spontaneous nystagmus and the spinning sensation (vertigo), mainly the decrease in resting discharge on the lesioned side. However, additional mechanisms contributing to acute asymmetry are possible. The ‘on-off’ response direction of vestibular afferents [23] show a minor asymmetry around the resting discharge with larger gain for ipsiversive rotations, which may play an additional role in enhancing the threshold asymmetry. Recordings from vestibular nuclei cells in healthy animals show fewer units responding to contralateral rotations (compared to ipsilateral rotations, [24]), with these units also showing higher resting discharge rates [28]. Reduced activity in off-direction units from the affected ear, coupled with increased activity in on-rotational type I units (resulting from loss of inhibitory drive from the affected ear via commissural connections) may lead to increased thresholds towards the healthy side observed in some patients acutely. The concurrent loss of excitatory responses from the lesioned peripheral afferents may additionally lead to a lower overall gain, increasing thresholds bilaterally, as suggested by the modelled responses.

In psychophysical terms, one might expect the perception of vertigo to add a strong physiological bias or perceptual “noise” into the VP system, impacting on its signal detection capabilities at the perceptual level specifically. Interestingly, however, we found that acute vertigo does not preferentially interfere with the cognitive process inherent to the perceptual task. This was shown, firstly, by the similar VO-VP threshold gap in patients and controls and, secondly, by the lack of any significant reduction in perceptual thresholds from acute to recovery stages (where vertigo is absent) in those patients with remaining large canal paresis (>70%). Vertigo appears to cause asymmetric thresholds, corresponding in our model to the decrease in resting discharge on the lesioned side. Indeed, the parallel VO and VP threshold findings, both experimentally and modelling, imply little or no impact of any additional processing by the cortex.

Thus, as with other sensory modalities, for example visual, auditory (pure tone audiometry), and tactile thresholds [29], VP threshold measurements essentially reflect the sensitivity of the peripheral receptor. Vestibulo-perceptual thresholds are argued to result from high-pass filtering at lower frequencies [30], similar to that of the VOR [30], [31]. Although additional neural processing involved in perceptual decision-making may increase perceptual thresholds above those of the VOR [6], [31]–[33], the agreement between human perceptual rotational thresholds and the sensitivity of primary afferents has been noted previously [34]. Our current findings show, a similar degree of asymmetry in VO and VP thresholds acutely, and no accentuation of the normal VO-VP gap in VN. Therefore we conclude that the decision criterion to generate a qualitative psychophysical response is elevated as compared to the reflex threshold, but any additional cortical processes occurring in acute VN are not apparent in threshold processing.

This conclusion is supported by the predictions of the threshold model, which assumes that the only difference between VP and VO threshold is the decision criterion requiring a larger separation of signal and noise for VP than for VO thresholds (see Methods and Results). An obvious limitation of the model is that several parameters have to be inferred from the experimental data, since they are not directly accessible. For example, we used the degree of acute canal paresis value as an estimate for the loss of peripheral afferent input. The acute canal paresis value was used not only for predicting acute threshold responses, but also for threshold recovery prediction, because recovery processes such as recalibration can conceal the remaining peripheral loss [35]. Another critical assumption of the model is that in the acute stage, the central fusion mechanism does not yet account for the increased variability of the lesioned afferent response by decreasing the weight of the ipsilesional afference. Alternatively, the central fusion mechanism may evaluate the instantaneous afferent variability by, for example, averaging over the deviation of each afferent input and the overall mean input. The latter is, however, more difficult to implement neuronally and we are not aware of physiological evidence for such a process. The model predictions for this case would change quantitatively only for the acute case, but would also predict an asymmetry of acute thresholds.

The second main finding is that, in contrast to threshold responses, supra-threshold responses show considerable VO-VP dissociation. Vestibulo-perceptual time constants were consistently reduced and symmetrical. The novel finding is that such remarkably symmetrical and bilateral suppression took place despite the recognised VOR asymmetry found in this and previous studies in acute unilateral vestibular failure [36]–[38]. This is indicative of additional, higher order processing of vestibular signals following unilateral vestibular loss.

The observed symmetrical reduction of perception of supra-threshold stimuli was observed at the acute stage only, critically when vertigo symptoms are maximal. We propose that this represents a perceptual compensatory mechanism - an overall dampening of supra-threshold vestibular perception as a result of the vertigo associated with a unilateral vestibular lesion. Various mechanisms might contribute to the dampening. A shortening of the time constant is expected to occur with increased uncertainty of the vestibular afferent input due to the velocity-storage mechanism [39], [40] - the increase in uncertainty is a consequence of loss of afferent nerve fibres (see model section). This mechanism, however, would apply similarly to both VO and VP systems and cannot explain the differential effects observed between VO and VP results.

Additional habituation-like mechanisms might contribute to the dampening. Vestibular habituation occurs when vestibular responses become shorter after repeated presentations of a stimulus. Vestibulo-ocular habituation is plane and direction specific [41] and repeated unidirectional vestibular stimulus exposures induce asymmetric shortening of VOR responses [42]. In contrast, results from vestibular perceptual habituation are mixed, with asymmetries in perception seemingly much harder to attain [42], [43]. Given that cortical circuits participate in habituation [44] and that the role of habituation is to protect against cortical overstimulation [45], habituation processes would offer a “protective barrier” in the context of intense vertigo due to asymmetrical peripheral vestibular input, thus providing an acute compensatory role.

Few studies have compared VO and VP responses and show conflicting results. Previous work has shown good correlation between VO-VP responses implying that vestibular perception of angular motion is driven by similar central processes as reflexive eye movements [10], [12], [46]. On the other hand, semicircular canal signals subserving perception appear to undergo differential central processing mechanisms [47]–[49] and here we present further evidence in support: a) the contrast between asymmetric VO and symmetric VP supra-threshold responses (Figures 4 and 5), b) the association between measures of peripheral vestibular function, such as caloric results and VOR gain, with supra-threshold VO but not VP responses and c) the absence of correlation between threshold and supra-threshold data in VP data despite a strong correlation between these variables for VO data (Figure 6). These findings therefore suggest additional neural processing for angular self-motion perception beyond the contribution of the brainstem velocity-storage mechanism of the VOR. Such additional processing may involve midline cerebellar regions [17] and indeed vestibulo-cerebello-thalamo-cortical pathways have been demonstrated [50]. Local processing by one or more of the many cortical areas known to receive vestibular projections is also possible [51], [52].

In conclusion, vestibulo-perceptual function is significantly affected by vestibular neuritis -VP thresholds are raised and asymmetrical whereas VP supra-threshold time constants are shortened but symmetrical. Acute VO and VP thresholds behave similarly and show a large degree of asymmetry – findings well described by a model assuming that the concurrent decrease in ipsilesional resting discharge and gain directly affects the central signal detection mechanism. In contrast to vestibular thresholds, there is dissociation between VP and VO responses to supra-threshold stimuli. Perceptual time constants are bilaterally reduced during supra-threshold stimuli – rotations that normally induce vertigo-like symptoms. The bilateral suppression of supra-threshold vestibular perception may act as a protective mechanism against vertigo. The perceptual dampening observed acutely provides the first functional evidence that higher-order mechanisms, ultimately involving the cerebral cortex, are engaged early in vestibular compensation and vertigo suppression.

## Supporting Information

Appendix S1 Sensor fusion and signal detection model.
(DOC)
