# Supplementary material for: Vestibular Perception following Acute Unilateral Vestibular Lesions
Source: PLoS One. 2013 May 9;8(5):e61862. doi: 10.1371/journal.pone.0061862 (PMC3650015; doi:10.1371/journal.pone.0061862)
Supplement: Appendix S1 — Sensor fusion and signal detection model. (DOC) [file pone.0061862.s001.doc]

**Appendix S1**

We assume that *n* peripheral neurons on one side of the vestibular system contribute to the central response. Each of the contributing neurons has a baseline firing rate *b* and a gain factor *g*, so that the firing rate *f* of each neuron is determined by *f*(*s*)=*b*+*g*∙*s*+ε with *s* being the stimulus and ε additive Gaussian noise with zero mean and variance σ^2^. Additionally, baseline firing rate *b* and gain *g* are normally distributed over the population of neurons.

The central response is formed by summation of the neurons’ firing rates *f* and division by the number of neurons *n*. This averaging corresponds to maximum-likelihood estimation; it reduces the noise and constitutes the best option for fusing the responses without additional assumptions [1]. The central response has then a firing rate of *f_c_*=*b*+*g*∙*s*+ε*_c_* with the variance of the central noise ε*_c_* being σ^2^/*n*. Such central fusion has been supported experimentally showing that psychophysical thresholds are lower than those of individual vestibular-nuclei neurons [2].

To simulate a partial lesion of the peripheral system, we assume that a fraction of neurons *c∙n* is destroyed (0≤*c*≤1) and their firing rate is set to zero. Without any central compensation, this leads to a firing rate at the central neuron, which is scaled down by a factor (1-*c*) in baseline firing and gain, but has a variance increased by a factor 1/(1-*c*). Thus, using these simplifying assumptions, we can now model the changes in gain, baseline firing, and noise with a single parameter, which is the fraction of neurons left on the lesioned side.

As mentioned in the methods, the response of the healthy side to a velocity stimulus *s* is the firing rate *f*(*s*)=*b*+*s.* The lesioned side will respond with

*f_l_*(*s*)=(1-*c*)∙(*b*+*s*) Eqn. 1

Since the firing may be already partly restored at the time of the experiment, we use *f_c_*=*b*+(‑*s*) for the healthy contralateral side and *f_i_*=(*b-*β)+(1-c)∙*s* for the lesioned ipsilateral side with β being the loss in ipsilateral firing. The fused response is thus

*f_f_*(*s*)=(*f_i_*-*f_c_*)/2=*-*β/2+(1-c/2)∙*s* Eqn. 2

From this equation it follows that for a stimulus *s*=0 the central response is *f*(0)=*-*β/2, which corresponds to a rotation to the contralateral side.

After recovery, the lesioned side responds with *f*(*s*)=*b*+*s* just like the healthy side, but with increased variability σ^2^/(1-*c*)*.* Accordingly, if the central maximum-likelihood estimate takes into account this change in variance, the resulting central estimate has now the increased variance σ^2^/(2-c).

Thus, for the normal case and the acute lesion, the threshold stimulus *s_t_* is determined by the equality

*f*(*s_t_*)=±*λ*∙σ/sqrt(2) Eqn. 3

with *f*(*s_t_*) being the central response determined by equation 2, *λ* being a factor, and σ being the standard deviation of the signal distribution (see Figure 2A). For the recovery case, this relation changes to

*f*(*s_t_*)=±*λ*∙σ/sqrt(2-c) Eqn. 4

In the healthy case, the three unknown variables are: the decision factor *λ*, the signal SD σ, and the amount of lesion *c*. For simplicity we assume that β is proportional to the acute spontaneous nystagmus. From equation 2 and 3 it follows that the threshold in the acute case is asymmetric and becomes

*s_ta_*=(±2*s_th_-*β)/(2-c) Eqn. 5a

with *s_th_* being the threshold of healthy subjects. For the recovery, the threshold becomes symmetric again with

*s_tr_*=±*s_th_*∙sqrt(2)/sqrt(2-c) Eqn. 5b

Reference List

1. Ernst M, Banks M (2002) Humans integrate visual and haptic information in a statistically optimal fashion. Nature 415: 429-433.

2. Massot C, Chacron MJ, Cullen KE (2011) Information transmission and detection thresholds in the vestibular nuclei: single neurons vs. population encoding. J Neurophysiol 105: 1798-1814. jn.00910.2010 [pii];10.1152/jn.00910.2010 [doi].
